# Association of Diabetes Mellitus With Increased Mortality in Carbapenem-Resistant Enterobacterales Infections

**DOI:** 10.7759/cureus.53606

**Published:** 2024-02-05

**Authors:** Mohamed Aon, Ahmed H Aoun, Ahmad Al Shami, Abdulrahman Alharbi, Khaled Aljenfawi, Sarah Al-Anazi, Fares Salman, Mohammed Assaf, Magd Mobarak, Ebtehal AlRoomi, Omar A Abdelwahab, Mohamed M Ibrahim

**Affiliations:** 1 Department of Internal Medicine, Faculty of Medicine, Cairo University, Giza, EGY; 2 Department of Pediatrics, Faculty of Medicine, Cairo University, Giza, EGY; 3 Department of Pediatrics, Primary Health Care Corporation, Doha, QAT; 4 Department of Internal Medicine, New Jahra Hospital, Jahra, KWT; 5 Department of Microbiology, New Jahra Hospital, Jahra, KWT; 6 Department of Internal Medicine, Faculty of Medicine, Al-Azhar University, Cairo, EGY; 7 Department of Internal Medicine, Jaber Al-Ahmed Armed Forces Hospital, Kuwait, KWT

**Keywords:** cre, mortality, enterobacterales, diabetes mellitus, carbapenem-resistant

## Abstract

Introduction

Carbapenem-resistant *Enterobacterales* (CRE) infections have high mortality. We aimed to examine the diabetes mellitus (DM) association with CRE mortality.

Methodology

Our study is a retrospective cohort study including patients who were admitted to the medical wards in the main district hospital (New Jahra Hospital, Kuwait) between January 1, 2022, and January 1, 2023, and diagnosed with CRE infections during hospitalization. The patients were divided into diabetic and non-diabetic groups. Clinical and laboratory data were collected. The presence of carbapenemase genes was detected. The primary outcome was 30-day hospital mortality. We assessed the effect of glycemic control on the outcomes.

Results

We included 47 patients in the diabetic group and 39 patients in the non-diabetic group. Females represented 54.7% of patients, and the median age was 73 and 55 years in the two groups, respectively. *Klebsiella pneumonia *(86%) and *Escherichia coli* (12.8%) were the most frequently isolated CRE. Carbapenemase genes were detected in all patients: NDM-1 in 67.4%, OXA-48 in 18.6%, and both genes coexisted in 14%. The 30-day hospital mortality was significantly higher in the diabetic group compared to the non-diabetic group (48.9% vs. 28.2%, P = 0.041). Among the diabetic patients, there was no significant difference between survivors and non-survivors regarding median glucose or glycated hemoglobin (HbA1c) levels (P = 0.465 and 0.932, respectively). Moreover, levels of glucose (odds ratio (OR) 0.928, confidence interval (CI) 0.763-1.13, P = 0.457) and HbA1c (OR 0.89, CI 0.63-1.26, P = 0.507) were not risk factors for increased mortality among diabetic patients.

Conclusion

We demonstrated the association between DM and increased CRE mortality regardless of the level of glycemic control. This study demonstrates the interaction between communicable and non-communicable diseases.

## Introduction

Carbapenem-resistant *Enterobacterales* (CRE) are defined as *Enterobacterales* that show resistance to one or more of the available carbapenem antibiotics or produce a carbapenemase. Resistance to carbapenems other than imipenem is required to define some *Enterobacterales* as CRE (e.g., *Providencia*, *Proteus*, and *Morganella*) because these bacteria have intrinsic resistance to imipenem [1]. CRE can cause serious healthcare-associated infections, such as bloodstream infections, hospital-acquired pneumonia, ventilator-associated pneumonia, intra-abdominal infections, and urinary tract infections [2]. CRE topped the World Health Organization’s list of highly resistant bacteria with the greatest threat to human health [3]. CRE can be divided into carbapenemase-producing CRE (CP-CRE) and non-carbapenemase-producing CRE (non-CP-CRE).

The CP-CRE are more virulent and associated with a poorer prognosis compared to non-CP-CRE [4]. CP-CRE clinically relevant carbapenemases include* Klebsiella pneumoniae* carbapenemase (KPC), New Delhi metallo-ß-lactamase-1 (NDM-1), Verona integron-encoded metallo-ß-lactamases (VIM), imipenem-resistant *Pseudomonas* (IMP)-type carbapenemase, and OXA-48-like carbapenemases [5]. Non-CP-CRE has different mechanisms that confer resistance to carbapenems, such as efflux pumps, porin loss, and the production of other β-lactamases that inactivate, rather than hydrolyze, carbapenems [6].

Several risk factors are associated with worse outcomes among CRE-infected patients, such as comorbidities (e.g., cancer, neutropenia, kidney disease, and cardiovascular diseases), higher clinical severity scores (e.g., Acute Physiology And Chronic Health Evaluation (APACHE) II score, Charlson index, and Sequential Organ Failure Assessment (SOFA) score), prior exposure to carbapenems, failure to control the source of infection, and inappropriate definitive therapy [7].

Diabetes mellitus (DM) was found to be associated with worse outcomes in some studies [7-9]. However, none of these studies were designed specifically to examine the impact of DM on the outcome of CRE infections. To our knowledge, no study was designed to examine the impact of DM on the outcome of CRE infections. This work aims to study the outcome of CRE infections in diabetic patients and to study any DM characteristics associated with worse outcomes.

## Materials and methods

Our study is a retrospective cohort study including patients who were admitted to the medical wards in the main district hospital (New Jahra Hospital, Kuwait) between January 1, 2022, and January 1, 2023, and diagnosed with CRE infections during hospitalization. The patients were divided into two groups according to the presence or absence of DM. Data from the hospital medical records were retrieved for demographic data, the Charlson Comorbidity Index (CCI) for comorbidities, and clinical, microbiological, and laboratory parameters on day 1 (the day on which the cultures were collected). Moreover, data on antimicrobial therapy were retrieved and analyzed to determine if the patients had received appropriate therapy. The therapy was considered appropriate if the CRE isolate was susceptible to at least one of the antibiotics given.

Patients were included in the study if they were hospitalized and had a positive culture showing CRE growth at a clinically significant site. Patients with polymicrobial bacterial infections, carbapenem-resistant *Acinetobacter baumannii*, or carbapenem-resistant *Pseudomonas aeruginosa* infections were excluded from the study. Specimens from pregnant females and patients aged <18 years were excluded.

Diabetes was identified if there was evidence in the patient’s medical record of DM diagnosis and/or glycated hemoglobin (HbA1c) ≥6.5% [10]. For all diabetic patients, HbA1c and serum glucose on day 1 were recorded. All patients were treated with a subcutaneous basal-bolus insulin regimen targeting a serum glucose level between 7.8 and 10 mmol/L.

CRE detection and antibiotic sensitivity testing were performed according to the Clinical and Laboratory Standards Institute (CLSI) standards. CRE was defined as isolates producing carbapenemases or isolates with a minimum inhibitory concentration (MIC) ≥4 μg/ml for carbapenems [11]. The molecular biological detection of carbapenemase-producing bacteria was done using the Eazyplex® SuperBug CRE amplification system (AmplexDiagnostics GMBH, Gars-Bahnhof, Germany). Procalcitonin (PCT) was measured using an automated enzyme-linked fluorescent assay (VIDAS® B.R.A.H.M.S. PCT™, BioMérieux Diagnostics, Inc., Lyon, France), and CRP was measured using the IMMAGE® Immunochemistry System (Beckman Coulter, Inc., California, USA). 

The primary outcome compared between the two groups was 30-day hospital mortality. Secondary outcomes included the improvement in the clinical condition, the need for intensive care unit (ICU) admission, and the improvement in the need for organ support. Clinical improvement was defined as improvement in sepsis score (Quick SOFA (qSOFA) score) between day 1 and day 7. The requirements of organ support, such as vasopressor drugs, renal replacement therapy (RRT), or ventilatory support, e.g., invasive mechanical ventilation (IMV)/non-invasive ventilation (BiPAP or CPAP) were recorded on days 1 and 7. Patients who required any of these organ support modalities were scored one point. The maximal score was three (i.e., a patient who requires vasopressor drugs, ventilatory support, and RRT). The improvement in organ support is equal to the score on day 1 minus the score on day 7. Improvement was present if the difference ≥1. Worsening was present if the difference was ≤-1. We also assessed the effect of the glycemic control measured by glucose and HbA1c levels on the outcomes.

The Jahra Hospital Research Committee issued approval J2-29012023. The need for informed consent was waived by the approving research committee due to the retrospective nature of the study. All patient data were anonymous. 

Sample size

The sample size was calculated using an expected outcome incidence of 43% [12] and an assumed relative risk of 1.7 [13] to achieve a study power of 80% with a confidence level of 95%. The calculated sample size was 39 patients in each group (a total of 78 patients).

Statistical analysis

Categorical variables were presented as absolute numbers and their percentages, while continuous variables were presented as mean and standard deviation (SD) or median and interquartile ranges (IQR), as appropriate. Data were compared between the groups using the Chi-square test or Fisher's exact test for categorical variables and the Student's t-test or Kruskal-Wallis test for quantitative variables, as appropriate. We considered P-value <0.05 as statistically significant. We performed a logistic regression to explore the relationship between different variables and increased 30-day mortality among diabetic patients. Initially, a univariate analysis was made. Then, all significant variables in the univariate analysis were included in the multivariate model and adjusted for other variables in a stepwise approach. The data were analyzed using IBM SPSS Statistics for Windows, version 26 (released 2019; IBM Corp., Armonk, New York, United States).

## Results

A total of 86 hospitalized patients who were diagnosed with CRE infections were included, 47 (54.7%) patients in the diabetic group and 39 (45.3%) patients in the non-diabetic group. Females represented 48.9% (23/47) of the patients in the diabetic group and 61.5% (24/39) of patients in the non-diabetic group. The diabetic patients tended to be older (median age was 73 vs. 55 years), have more comorbidities (median CCI was 7 vs. 3 points), and have higher serum creatinine and glucose levels on day one (162 vs. 73 umol/L and 9.7 vs. 5.8 mmol/L, respectively) as compared to the non-diabetic group. At baseline, there was no significant difference between the groups as regards the qSOFA score or the organ support needs. The baseline clinical and laboratory characteristics of both groups are shown in Table 1. 

**Table 1:** Baseline demographic, clinical, and laboratory characteristics. ^a ^Data are presented as median (IQR) or number (%). ^b^ Numbers refer to the number of modalities of organ support needed by the patient e.g., ventilatory support, RRT, and/or vasopressor drugs (details in the Methods section). Abbreviations: ALT, alanine transaminase; AST, aspartate aminotransferase; CCI, Charlson Comorbidity Index; CRP, C-reactive protein; IQR, interquartile range; PCT, procalcitonin; RRT, renal replacement therapy; WBCs, white blood cells

Variable ^a^	Diabetic	Non-diabetic	p-value
	(n = 47)	(n = 39)	-
Age (years)	73 (64, 84)	55 (36, 78)	<0.001
Sex	-	-	0.243
Female	23 (48.9%)	24 (61.5%)	-
Male	24 (51.1%)	15 (38.5%)	-
CCI	7.0 (5.0, 8.5)	3.0 (0.0, 6.0)	<0.001
qSOFA (Day 1)	-	-	0.617
0	4 (8.5%)	5 (12.8%)	-
1	24 (51.1%)	23 (59.0%)	-
≥ 2	19 (40.4%)	11 (28.2%)	-
Organ support needs on day1^b^	-	-	0.682
0	15 (31.9%)	16 (41.0%)	-
1	13 (27.7%)	12 (30.8%)	-
2	16 (34.0%)	9 (23.1%)	-
3	3 (6.4%)	2 (5.1%)	-
CRP (mg/dl)	10 (5, 22)	10 (2, 17)	0.282
PCT (ng/ml)	1 (0, 4)	2 (0, 7)	0.116
WBCs (*10^9^/L)	10 (8, 15)	9 (6, 15)	0.328
Neutrophils (*10^9^/L)	8 (6, 13)	7 (4, 13)	0.596
Lymphocytes (*10^9^/L)	1.30 (0.78, 2.20)	1.27 (0.90, 1.92)	0.967
Hemoglobin (g/L)	86 (75, 98)	93 (86, 103)	0.088
Platelets (*10^9^/L)	224 (115, 277)	234 (127, 365)	0.308
Creatinine (umol/L)	162 (72, 287)	73 (39, 140)	0.007
AST (IU/L)	30 (16, 51)	20 (13, 40)	0.111
ALT (IU/L)	18 (11, 36)	14 (9, 24)	0.195
Glucose (mmol/L)	9.70 (7.55, 11.30)	5.80 (4.82, 6.77)	<0.001

Table 2 demonstrates the microbiological characteristics of our cohort. The most frequently isolated CRE was *Klebsiella pneumonia* in 74 patients (86%), followed by *Escherichia coli* in 11 patients(12.8%) and *Enterobacter cloacae* in one patient (1.2%), without significant differences between the groups. CRE was detected mainly in respiratory samples (n = 25, 29.1%), blood (n = 23, 26.7%), and urine cultures (n = 20, 23.3%). Carbapenemase genes were detected in all patients: NDM-1 in 58 patients (67.4%), OXA-48 in 16 patients (18.6%), and both genes coexisted in 12 patients (14%). A second culture result to confirm microbiological eradication after seven days of antimicrobial therapy was available only for 36/86 patients (41.9%). Only three patients (3.5%) achieved microbiological eradication with no significant difference between the groups. There was no difference between the groups as regards the administration of appropriate antimicrobial therapy or use of combination therapy (P = 0.954 and 0.530, respectively). Thirty-four patients received monotherapy, and the majority of them (n = 22, 64.7%) received colistin monotherapy.

**Table 2:** Microbiological characteristics. ^a^ P-value when comparing the diabetic and the non-diabetic groups. ^b^ Respiratory sample includes sputum, ETT, and BAL cultures. ^c^ Other cultures include wound swabs (n = 14), discharges (n = 2), and fluid cultures (n = 2). ^d^ CRE isolate is susceptible to at least one of the antibiotics used. Abbreviations: BAL, bronchoalveolar lavage; CZA, ceftazidime/avibactam; *E. cloacae*, *Enterobacter cloacae*; *E. coli*,* Escherichia coli*; ETT, endotracheal tube; *K. pneumonia*, *Klebsiella pneumonia*; TMP-SMX, trimethoprim-sulfamethoxazole

	Diabetics (n = 47)	Non-diabetics (n = 39)	Total (n = 86)	P-value ^a^
Culture organism				0.218
K. pneumonia	43.0 (91.5%)	31.0 (79.5%)	74.0 (86.0%)	
E. coli	4.0 (8.5%)	7.0 (17.9%)	11.0 (12.8%)	
E. cloacae	0.0 (0.0%)	1.0 (2.6%)	1.0 (1.2%)	
Culture site				0.254
Blood	14.0 (29.8%)	9.0 (23.1%)	23.0 (26.7%)	
Respiratory^ b^	15.0 (31.9%)	10.0 (25.6%)	25.0 (29.1%)	
Urine	7.0 (14.9%)	13.0 (33.3%)	20.0 (23.3%)	
Other^ c^	11.0 (23.4%)	7.0 (17.9%)	18.0 (20.9%)	
Antibiotic sensitivity				
Amikacin	29.0 (61.7%)	30.0 (76.9%)	59.0 (68.6%)	0.130
Gentamicin	28.0 (59.6%)	25.0 (64.1%)	53.0 (61.6%)	0.667
Colistin	35.0 (74.5%)	19.0 (48.7%)	54.0 (62.8%)	0.014
Fosfomycin	7.0 (14.9%)	7.0 (17.9%)	14.0 (16.3%)	0.702
Tigecycline	23.0 (48.9%)	19.0 (48.7%)	42.0 (48.8%)	0.984
TMP-SMX	2.0 (4.3%)	1.0 (2.6%)	3.0 (3.5%)	0.670
CZA	3.0 (6.4%)	3.0 (7.7%)	6.0 (7.0%)	0.812
Antibiotic treatment				
Amikacin	10.0 (21.3%)	15.0 (38.5%)	25.0 (29.1%)	0.081
Gentamicin	3.0 (6.4%)	2.0 (5.1%)	5.0 (5.8%)	0.804
Colistin	36.0 (76.6%)	21.0 (53.8%)	57.0 (66.3%)	0.026
Fosfomycin	6.0 (12.8%)	5.0 (12.8%)	11.0 (12.8%)	0.994
Tigecycline	21.0 (44.7%)	22.0 (56.4%)	43.0 (50.0%)	0.279
CZA	3.0 (6.4%)	3.0 (7.7%)	6.0 (7.0%)	0.812
Meropenem	4.0 (8.5%)	2.0 (5.1%)	6.0 (7.0%)	0.540
Combination therapy				0.530
No	20.0 (42.6%)	14.0 (35.9%)	34.0 (39.5%)	
Yes	27.0 (57.4%)	25.0 (64.1%)	52.0 (60.5%)	
Appropriate therapy^ d^	42.0 (89.4%)	35.0 (89.7%)	77.0 (89.5%)	0.954
Genotype				0.834
NDM-1	33.0 (70.2%)	25.0 (64.1%)	58.0 (67.4%)	
OXA-48	8.0 (17.0%)	8.0 (20.5%)	16.0 (18.6%)	
NDM+OXA48	6.0 (12.8%)	6.0 (15.4%)	12.0 (14.0%)	
Microbiology clearance				0.357
Yes	1.0 (2.1%)	2.0 (5.1%)	3.0 (3.5%)	
No	21.0 (44.7%)	12.0 (30.8%)	33.0 (38.4%)	
Unknown	25.0 (53.2%)	25.0 (64.1%)	50.0 (58.1%)	

**Figure 1:**
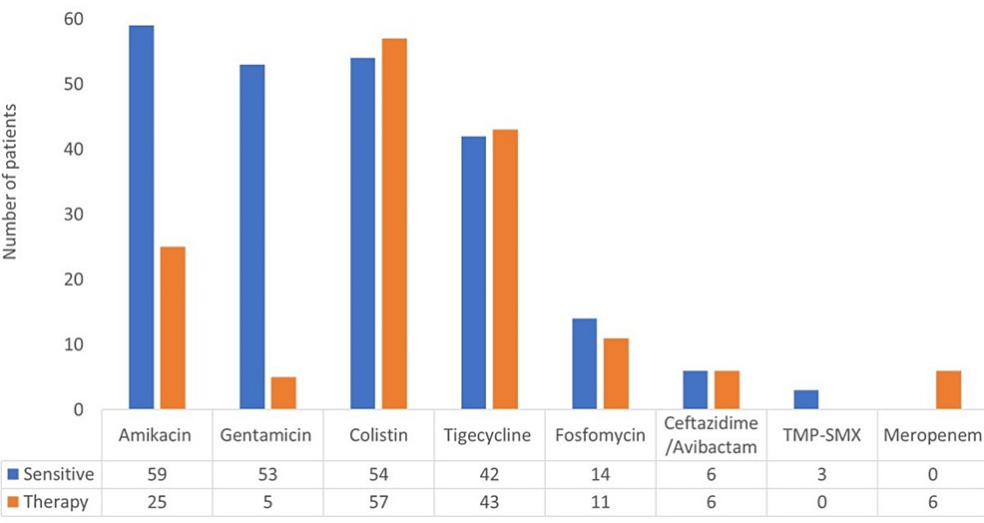
Antibiotics sensitivity and therapy. The figure compares the patterns of prescription of each antibiotic as compared to its in vitro sensitivity patterns. Abbreviations: CZA, ceftazidime/avibactam; TMP-SMX, trimethoprim-sulfamethoxazole

Aminoglycosides, colistin, and tigecycline were the most frequently observed antibiotics with in vitro activity against CRE. The antibiotic sensitivity and prescription patterns are demonstrated in Figure 1.

The 30-day hospital mortality was higher in the diabetic group (n = 23, 48.9%) compared to the non-diabetic group (n = 11, 28.2%), and the difference was statistically significant (P = 0.041). More diabetic patients required ICU admission (n = 30, 63.8%) as opposed to the non-diabetic group (n = 22, 56.4%), but the difference was not statistically significant (P = 0.316). There was no substantial difference between the two groups concerning improvement in sepsis score or organ support needs (P = 0.640 and 0.255, respectively), as shown in Table 3. 

**Table 3:** Clinical outcomes by diabetes status. ^a^ P-value when comparing the diabetic and the non-diabetic groups. ^b^ Clinical improvement means improvement in qSOFA score between day 7 and day 1. ^c^ Numbers refer to the number of modalities of organ support needed by the patient, e.g., ventilatory support, RRT, and/or vasopressor drugs (details in the Methods section). Abbreviations: ICU, intensive care unit; qSOFA, quick SOFA score; RRT, renal replacement therapy

	Diabetics (n = 47)	Non-diabetics (n = 39)	Total (n = 86)	p-value ^a^
qSOFA (day 7)				0.348
0	9.0 (19.1%)	11.0 (28.2%)	20.0 (23.3%)	
1	28.0 (59.6%)	24.0 (61.5%)	52.0 (60.5%)	
≥2	10.0 (21.3%)	4.0 (10.3%)	14.0 (16.3%)	
Clinical improvement ^b^				0.640
Yes	18.0 (38.3%)	14.0 (35.9%)	32.0 (37.2%)	
No change	24.0 (51.1%)	23.0 (59.0%)	47.0 (54.7%)	
Worsen	5.0 (10.6%)	2.0 (5.1%)	7.0 (8.1%)	
Organ support needs on day 7 ^c^				0.160
0	15.0 (31.9%)	21.0 (53.8%)	36.0 (41.9%)	
1	12.0 (25.5%)	9.0 (23.1%)	21.0 (24.4%)	
2	13.0 (27.7%)	7.0 (17.9%)	20.0 (23.3%)	
3	7.0 (14.9%)	2.0 (5.1%)	9.0 (10.5%)	
Improvement in organ support needs				0.255
Yes	6.0 (12.8%)	8.0 (20.5%)	14.0 (16.3%)	
No change	34.0 (72.3%)	29.0 (74.4%)	63.0 (73.3%)	
Worsen	7.0 (14.9%)	2.0 (5.1%)	9.0 (10.5%)	
ICU admission				0.316
Yes	30.0 (63.8%)	22.0 (56.4%)	52.0 (60.5%)	
No	17.0 (36.2%)	17.0 (43.6%)	34.0 (39.5%)	
30-day mortality				0.041
Yes	23.0 (48.9%)	11.0 (28.2%)	34.0 (39.5%)	
No	24.0 (51.1%)	28.0 (71.8%)	52.0 (60.5%)	

Among the diabetic group, when we analyzed the diabetes characteristics in survivor patients (n = 24, 51%) and non-survivor patients (n = 23, 49%), we did not find a significant difference. The median (IQR) glucose level in the survivors’ group was 9.75 (7.88, 11.12) mmol/L, and in the non-survivor group, it was 8.90 (7.45, 11.40) mmol/L, and the difference was not significant (P = 0.465). Similarly, the median (IQR) HbA1c percentage was 7.95 (7.35, 8.77) among the survivors and 8.40 (6.80, 9.05) among the non-survivors, and the difference was not significant (P = 0.932). In addition, when the diabetic patients were divided into two groups based on the HbA1c level, there was no difference between the groups as regards clinical improvement, improvement in organ support needs, need for ICU admission, or 30-day mortality, as shown in Table 4.

**Table 4:** Clinical outcomes in diabetic patients by the HbA1c level. ^a^ P value when comparing the higher and the lower HbA1c diabetic groups. ^b^ Clinical improvement means improvement in qSOFA score between day 7 and day 1. Abbreviations: HbA1c, glycated hemoglobin; ICU, intensive care unit; qSOFA, quick SOFA score

	HbA1c ≤ 8% (n = 22)	HbA1c > 8% (n = 25)	All diabetics (n = 47)	P-value^ a^
Clinical improvement ^b^				0.585
Yes	7.0 (31.8%)	11.0 (44.0%)	18.0 (38.3%)	
No change	13.0 (59.1%)	11.0 (44.0%)	24.0 (51.1%)	
Worsen	2.0 (9.1%)	3.0 (12.0%)	5.0 (10.6%)	
Improvement in organ support needs				0.135
Yes	5.0 (22.7%)	1.0 (4.0%)	6.0 (12.8%)	
No change	15.0 (68.2%)	19.0 (76.0%)	34.0 (72.3%)	
Worsen	2.0 (9.1%)	5.0 (20.0%)	7.0 (14.9%)	
ICU admission	14.0 (63.6%)	16.0 (64.0%)	30.0 (63.8%)	0.979
30-day mortality	9.0 (40.9%)	14.0 (56.0%)	23.0 (48.9%)	0.302

The analysis of risk factors associated with the 30-day mortality among the diabetic patients is shown in Table 5. In the univariable analysis, higher CRP and PCT were risk factors for mortality, while glycemic parameters, e.g., glucose and HbA1c levels were not. In the multivariable model adjusted for covariates, higher CRP (OR 1.29, 95% CI 1.04-1.6, P = 0.020) and PCT (OR 1.50, 95% CI 1.06-2.12, P = 0.022) remained as independent risk factors for the 30-day mortality among the diabetic patients.

**Table 5:** Univariate and multivariate logistic regression for the association between 30-day mortality and the potential risk factors among diabetic patients. ^a^ Adjusted for culture site, organism, and genotype. Abbreviations: CCI, Charlson Comorbidity Index; CI, confidence interval; CRP, C-reactive protein; HbA1c, glycated hemoglobin; PCT, procalcitonin; WBCs, white blood cells

	Univariate analysis		Multivariate analysis ^a^	
Variables	Odds ratio (CI 95%)	P-value	Odds ratio (CI 95%)	P-value
Age	0.97 (0.93–1.02)	0.235	-	-
Sex (male vs. female)	3.13 (0.95–10.29)	0.061	-	-
CCI	1.03 (0.83–1.27)	0.785	-	-
Glucose	0.928 (0.763–1.13)	0.457	-	-
HbA1c	0.89 (0.63–1.26)	0.507	-	-
CRP	1.27 (1.07–1.51)	0.007	1.29 (1.04–1.6)	0.020
PCT	1.33 (1.01–1.74)	0.039	1.50 (1.06–2.12)	0.022
WBC	1.09 (0.98–1.21)	0.117	-	-
Neutrophils	0.99 (0.94–1.03)	0.579	-	-
Lymphocytes	0.85 (0.66–1.11)	0.238	-	-
Hemoglobin	0.99 (0.96–1.02)	0.346	-	-
platelets	0.996 (0.991–1.00)	0.163	-	-
Creatinine	1.003 (0.999–1.01)	0.173	-	-
Combination therapy	1.88 (0.85–6.06)	0.294	-	-

## Discussion

In this study, diabetes was associated with increased mortality among patients with CRE infections. The burden of CRE infection morbidity and mortality is well-known and is often attributed to factors related to the patient, treatment regimen, and the pathogen [12]. Carbapenem resistance has a major impact on the mortality of patients infected with *Enterobacterales*. Mortality is doubled with CRE infections compared to CSE [12], and the prognosis is worse with CP-CRE in comparison to non-CP-CRE [4]. In our cohort, all isolated organisms were CP-CRE, and the most frequently identified genotypes were NDM-1, OXA-48, or both genes coexpressed. Other carbapenemase genes, e.g., KPC and VIM, were not found in our cohort.

Previous studies from our region identified a similar pattern of carbapenemase genotypes [14]. Another study from Kuwait found that the most prevalent gene among CRE-infected patients was OXA-48 (32.3%), followed by NDM-1 (15.4%) and KPC (12.3%) [15]. The authors also reported that 35.4% of patients had dual genes and 4.6% of patients had triple genes. The presence of more than one genotype in a patient infected with CRE is associated with a higher mortality [16]. The current guidelines recommend carbapenemase gene testing to guide the ideal antimicrobial therapy choices [17]. Furthermore, the increased mortality observed with CRE may be partially explained by the incorrect initial antimicrobial therapy and the delay in proper therapeutic interventions [12].

Among our cohort, the most used antibiotics to treat CRE infections were colistin, tigecycline, and aminoglycosides. Although colistin is not the preferred agent to treat CRE infections according to the latest US guidelines [17], the different phenotypic and genotypic profiles in our region may justify its use provided that in vitro activity against CRE has been displayed. Most of our cohort CRE isolates were CP-CRE with NDM-1 genotype contrary to the US data [18]. In the era preceding the availability of newer antibiotics, colistin was frequently used as monotherapy or as a part of combination regimens [19]. Colistin monotherapy was found to be effective in treating CRE infections in the OVERCOME trial [20], and colistin-based combination regimens were associated with better outcomes [7]. Hence, colistin is still needed for the treatment of CRE infections given the limited availability and high cost of the newer antibiotics.

Aminoglycosides and tigecycline are other options for the treatment of CRE infections. However, tigecycline should not be used to treat bloodstream or urinary tract infections. A feature of tigecycline worth mentioning is that its activity is unrelated to the genetic type of carbapenemases [17]. Many of the isolated CRE in our study were sensitive to aminoglycosides, but the prescription of aminoglycosides showed a much lower rate. This can be explained by the safety concerns associated with their use, especially nephrotoxicity. Newer dosing strategies are less toxic compared to the standard multiple-dosing schedules [21]. The newer aminoglycoside, plazomicin, may be more effective in treating CRE isolates resistant to other aminoglycosides [22]. While ceftazidime/avibactam is recommended for the treatment of CRE infections, it was not widely used in our cohort due to its limited availability. Of note, ceftazidime-avibactam and aztreonam combination therapy is the preferred regimen to treat NDM-producing CRE [17].

DM is associated with a higher incidence of bacterial infections, such as urinary, respiratory, skin, and soft tissue infections [23]. Some bacterial infections, such as invasive otitis externa, emphysematous pyelonephritis, and cholecystitis, are seen exclusively in diabetic patients [24-26].

The main pathophysiological mechanisms explaining the interaction between hyperglycemic and bacterial infections include disordered humoral and cellular immunity, impaired secretion of inflammatory cytokines, imbalanced oxidant/antioxidant response, increased cellular apoptosis, the boosting effect of hyperglycemic on the growth and virulence of some pathogens, and the local deleterious effects of hyperglycemic on tissues blood and nerve supply due to diabetic microvascular changes [27,28]. Furthermore, studies have demonstrated the association between DM and increased hospitalization (HR 1.59, CI 1.52-1.67) and mortality (HR 1.71, CI 1.36-2.16) due to infections [29]. The current guidelines focus on treating hyperglycemia and its related traditional complications, i.e., microvascular and macrovascular complications. This was reflected in the rates of hospitalization, where admissions due to diabetes-specific causes declined in contrast to conditions not specific to DM, such as infections, which showed an up-trending admission rate [30]. The current standards of care for diabetic patients do not result in a decline in hospitalization due to infectious causes, which emphasizes the need for additional measures regarding prevention and treatment [31]. 

The results of earlier studies on* Enterobacteriaceae* infections were contradictory as regards the relationship between diabetes and increased mortality. Some found an association with increased mortality [32], while others did not [33]. The authors hypothesized that better glycemic control in diabetic patients may explain the discordance between the results, although antidiabetic medication details or hyperglycemia parameters were not assessed [33]. Moreover, these findings were among patients who had CSE.

A study from Taiwan revealed that diabetic patients who had CRE infections had approximately threefold higher mortality compared to non-diabetic (OR 3.56, CI 1.05-12.05, P = 0.04) [8]. Another study from Italy showed that DM was an independent risk factor for mortality (OR 1.56, 95% CI 1.07-2.27, P = 0.01) [9]. In these studies, DM was identified among other risk factors, and it was not intended to examine the effect of DM on CRE mortality by itself. A meta-analysis identified DM, among other comorbidities, associated with higher mortality in CRE-infected patients [7]. Nonetheless, no studies have focused on examining DM and its features associated with worse CRE infection outcomes.

Our results highlight the association between diabetic status and increased mortality among CRE-infected patients. However, among diabetic patients, different levels of glycemic control were not associated with differences in mortality. Our logistic regression model showed that among diabetic patients, glucose and HbA1c levels were not risk factors for mortality. The relation between glycemic control and infection remains a partially answered question. Observational studies examining the association between higher HbA1c with increased infection risk and mortality revealed contradictory results [34]. The results were also different between low-income and high-income settings [34]. We classified patients based on HbA1c level ≤ or >8%, which is a target appropriate for hospitalized frail patients with multiple comorbidities [10]. A recent study found an association between increased infection-related mortality and markedly elevated HbA1c levels ≥11%. The association was mainly noticed in death from soft tissue and skin infections [35].

This study, to the best of our knowledge, is the first to explore the effect of diabetes status on the outcome of CRE infections. It has demonstrated the association between DM status and increased mortality. Nevertheless, it has some limitations, such as the small sample size and the retrospective observational nature. Second, the organ support outcome did not have enough details on the oxygen therapy modalities. Moreover, other outcomes, such as microbiological eradication, were not fully reported due to data unavailability. Finally, the impact of intervention to treat hyperglycemia on the outcomes was not examined.

## Conclusions

Our study demonstrated that DM is associated with higher mortality among CRE-infected patients. Furthermore, we found that the presence of diabetes per se, regardless of the level of glycemic control, is associated with worse outcomes. This highlights the interaction between communicable and non-communicable diseases and emphasizes that diabetic patients with CRE infections need greater focus. In addition to hyperglycemic treatments according to standards of care, more attention should be paid to the early initiation of appropriate antimicrobial therapy. More studies are needed to offer comprehensive data on this subject. The impact of treating hyperglycemia on outcomes needs further research, preferably through randomized trials. Standards of care for diabetic patients should pay more attention to the management of infections and emphasize the early initiation of antibiotics upon clinical suspicion and appropriate use of molecular techniques to rapidly identify multi-drug resistant bacteria. We advise combining clinical assessment and serum biomarkers (CRP and PCT) to identify patients who are at risk of a poorer prognosis.
